# Prognostic analysis of patients with breast cancer based on tumor mutational burden and DNA damage repair genes

**DOI:** 10.3389/fonc.2023.1177133

**Published:** 2023-06-07

**Authors:** Xu Teng, Tianshu Yang, Baowen Yuan, Yunkai Yang, Jiaxiang Liu, Xin Wang, Yong Wang, Tianyu Ma, Xin Yin, Hefen Yu, Shuang Wang, Wei Huang

**Affiliations:** ^1^ Beijing Key Laboratory of Cancer Invasion and Metastasis Research, Department of Biochemistry and Molecular Biology, School of Basic Medical Sciences, Capital Medical University, Beijing, China; ^2^ Key Laboratory of Cancer and Microbiome, State Key Laboratory of Molecular Oncology, National Cancer Center/National Clinical Research Center for Cancer/Cancer Hospital, Chinese Academy of Medical Sciences and Peking Union Medical College, Beijing, China; ^3^ Department of Breast Surgical Oncology, National Cancer Center/National Clinical Research Center for Cancer/Cancer Hospital, Chinese Academy of Medical Sciences and Peking Union Medical College, Beijing, China; ^4^ Department of Ultrasound, National Cancer Center/National Clinical Research Center for Cancer/Cancer Hospital, Chinese Academy of Medical Sciences and Peking Union Medical College, Beijing, China; ^5^ Department of Cardio Surgery Center, Shandong Second Provincial General Hospital, Jinan, China

**Keywords:** breast cancer, TMB, cox regression analysis, Cox-LASSO regression analysis, prognostic model, tumor-infiltrating immune cells

## Abstract

**Background:**

Breast cancer has a high tumor-specific death rate and poor prognosis. In this study, we aimed to provide a basis for the prognostic risk in patients with breast cancer using significant gene sets selected by analyzing tumor mutational burden (TMB) and DNA damage repair (DDR).

**Methods:**

Breast cancer genomic and transcriptomic data were obtained from The Cancer Genome Atlas (TCGA). Breast cancer samples were dichotomized into high- and low-TMB groups according to TMB values. Differentially expressed DDR genes between high- and low-TMB groups were incorporated into univariate and multivariate cox regression model to build prognosis model. Performance of the prognosis model was validated in an independently new GEO dataset and evaluated by time-dependent ROC curves.

**Results:**

Between high- and low-TMB groups, there were 6,424 differentially expressed genes, including 67 DDR genes. Ten genes associated with prognosis were selected by univariate cox regression analysis, among which seven genes constituted a panel to predict breast cancer prognosis. The seven-gene prognostic model, as well as the gene copy numbers are closely associated with tumor-infiltrating immune cells.

**Conclusion:**

We established a seven-gene prognostic model comprising *MDC1*, *PARP3*, *PSMB1*, *PSMB9*, *PSMD2*, *PSMD7*, and *PSMD14* genes, which provides a basis for further exploration of a population-based prediction of prognosis and immunotherapy response in patients with breast cancer.

## Introduction

Breast cancer is one of the most common malignant tumors occurring in women. In recent years, the incidence of breast cancer has increased annually and has gradually become a veritable health risk for women (1). In 2020, approximately 2.3 million new cases of breast cancer occurred, accounted for 24.5% of all women cancer cases; approximately 682,000 deaths were due to breast cancer, accounted for 15.5% of all the women cancer-related deaths worldwide (2). Because of the high tumor-specific death rate of breast cancer, the prognosis of patients with breast cancer should be investigated. Currently, traditional clinical and pathological staging cannot effusively mirror tumor heterogeneity and predict the prognosis (3). With the development of cDNA microarray, high-throughput sequencing technology, along with methods for multiomics data integration, prediction models based on the combination of gene sequencing data and clinical data have gained considerable attention for the diagnosis and treatment of breast cancer (4, 5).

Tumor mutational burden (TMB) is defined as the total number of somatic gene coding errors, base substitutions, and gene insertion or deletion errors detected per million bases (6). Mutations are recognized by T cells, subsequently activating the immune response (7). Thus, TMB can reflect the curative effect of therapy to a certain extent. TMB, particularly microsatellite instability, is related to programmed death (PD) ligand 1 (PD-L1) levels in cancer cells. Moreover, the accumulation of mutations in the tumor genome can result in the translation of abnormal proteins through mutated mRNAs, leading to the production of new antigens and the presentation of new human leukocyte antigen (HLA) complexes in tumor cells (8). Therefore, the TMB index has been permitted by the Food and Drug Administration (FDA) for use in predicting the efficacy of pan-tumor immunotherapy. TMB is also closely associated with the expression of PD-1 and PD-L1, affecting the response to immunotherapy. Thus, the higher the TMB, the more likely the tumor cells would be discerned by the immune system and the higher the probability of immunotherapy efficacy (9, 10). Therefore, optimizing the algorithm for distinguishing high-and low-risk groups using TMB and improving the differentiation of patients with respect to adaptive immunotherapy response must be the focus of future research.

The DNA damage repair (DDR) pathway is vital in ensuring the accurate transmission of genetic material. Changes in the DDR pathway play a predictive and prognostic role in anticancer therapy (11). The occurrence and development of tumors are associated to abnormalities in the DDR pathway, such as the mutations in the homologous recombination repair (HRR) gene *BRCA1/2* in breast cancer (12). Approximately 10% of breast cancer cases occur in patients with germline pathogenic variants of *BRCA1*, *BRCA2*, and other DDR genes, which are correlated with an increased risk of breast, and other cancers. Studies have shown that DDR-related gene mutations are significantly correlated with TMB and that these genes can improve immunotherapy efficacy, which is associated with favorable outcomes (13). Hence, the establishment of a DDR gene panel based on TMB is crucial in optimizing the benefits and improving the therapeutic effects of immunologic agents, such as immune checkpoint inhibitors (ICIs), in patients with breast cancer.

Previous studies have used RNA sequencing to directly screen all differential genes and obtain the gene set related to breast cancer prognosis or to analyze the TMB of each tumor and predict the biomarkers associated with immunotherapy and TMB (14–19). In this study, we used breast cancer genomic and transcriptomic data and calculated TMB and to construct a breast cancer prognostic model based on differentially expressed DDR genes between high- and low-TMB groups and to determine potential biomarkers related to breast cancer prognosis, ultimately providing a theoretical reference value for the prognostic risk of patients with breast cancer. By analyzing the breast cancer genomic and transcriptomic data from The Cancer Genome Atlas (TCGA), we preliminarily identified seven DDR genes associated with a high TMB, constructed a prognostic model based on TMB and the DDR genes, and verified the model using the GSE26085 dataset from the Gene Expression Omnibus. We expect that our findings will provide a comprehensive basis for further exploration of a population-based prediction of prognosis and immunotherapy response in patients with breast cancer.

## Methods

### Genome and transcriptome data

**Figure 1:**
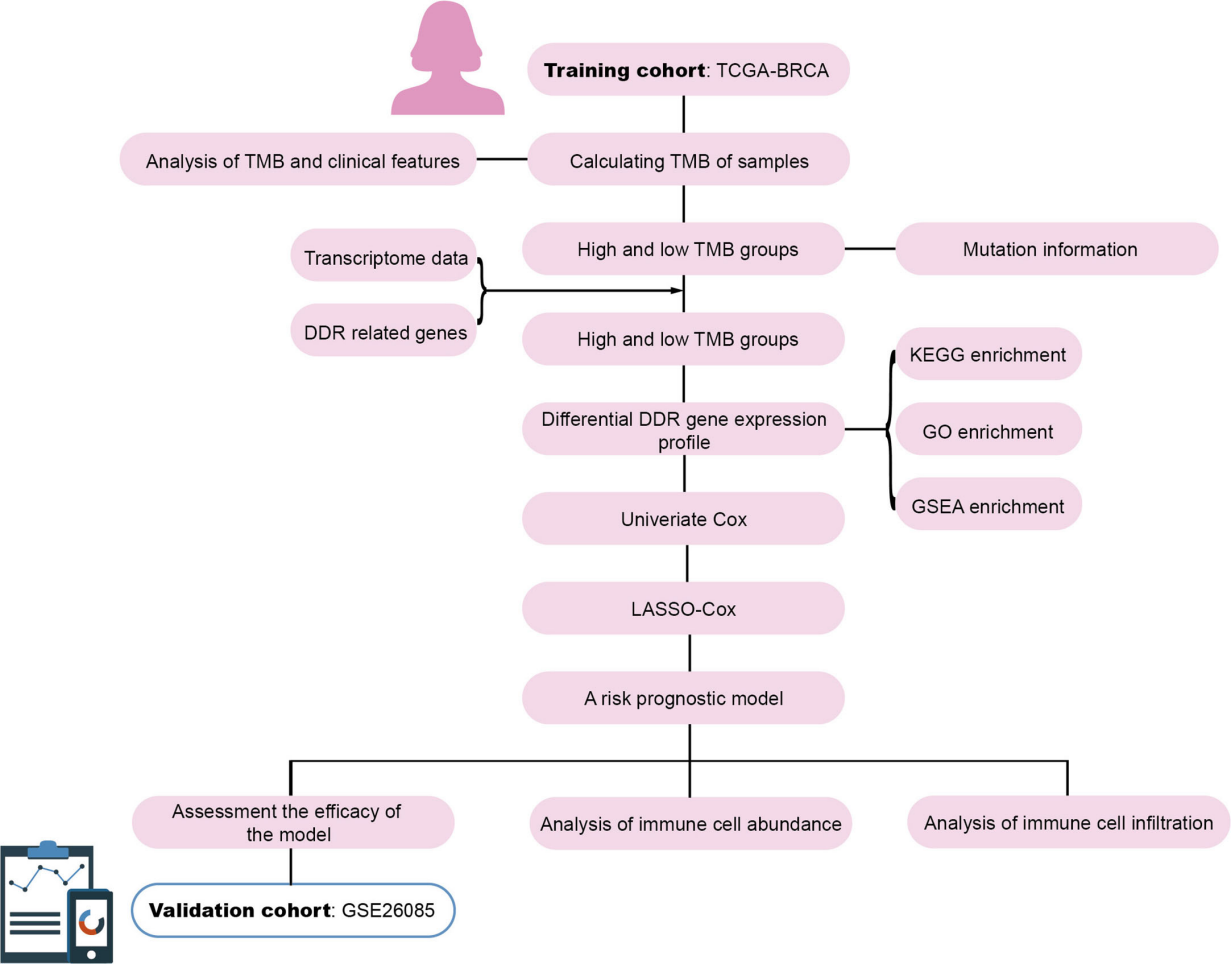
Workflow of the establishment of the prognostic model for breast cancer.

TCGA Breast cancer (BRCA) genomic and transcriptomic data were obtained from UCSC Xena (https://xenabrowser.net/). Among the 1,218 samples, samples without survival status (alive/dead) and overall survival (OS) time were excluded, the remaining 960 samples with both genomic and transcriptomic data were retained. The clinical information for each sample is presented in **Supplementary Table 1**. The GSE20685 dataset from GEO served as the validation cohort. The workflow is illustrated in **Figure 1**.

### Tumor mutational burden

TMB value of each sample was calculated using the TMB formula proposed by Lindsay Angus. et al. (20). The mutation rate per megabase (Mb) of genomic DNA was calculated as the total genome-wide amount of SNV, MNV and InDels divided over the total amount of mappable nucleotides (ACTG) in the human reference genome (hg19) FASTA sequence file. Each sample was dichotomized into high- or low-TMB group according to the median of TMB value.

### DDR gene sets and mutational landscape

DDR gene sets were collated as previously mentioned (21). DDR gene sets constitute 193 genes and 10 DDR pathway, involving like base-excision, nucleotide excision and mismatch repair for handling single-strand breaks, or homologous recombination repair, homology directed repair, non-homologous end joining, and Fanconi anemia pathways for handling double-strand breaks in DNA. The R package “maftools” was used to depict the DDR mutational landscape of the high- and low-TMB groups (22).

### Analysis of differential DDR gene expression profile

Differentially expressed DDR genes in the high- and low-TMB groups were screened according to |log2 (FC)| > 0.26 (i.e., 1.2-fold differential expression) and *P*-value < 0.05 (23, 24). DDR genes were intersected to obtain differentially expressed DDR genes using the “edgeR” R package (25).

### Enrichment analyses and signaling pathway analysis of DDR genes

KEGG analysis and GO functional enrichment analysis of the differentially expressed DDR genes was performed using the “clusterProfiler” R package (26). Signaling pathway analysis of the differentially expressed DDR genes was conducted using the GSEA software.

### Construction and assessment of prognostic model

For the differentially expressed DDR genes, univariate Cox proportional hazards regression analysis was used to select genes associated with prognosis using *P*-value < 0.05 as a filtering condition. The LASSO-Cox regression model was also used to select genes related to prognosis, and the correlation coefficients of these genes were obtained to construct a prognostic model. The risk score for each patient was determined according to the model, and the median of the risk scores served as the cutoff value for dividing the patients into high-and low-risk groups. The time-dependent ROC curves were used to evaluate the ability of the model to predict the 5-year and 10-year survival rates. The survival curves of the high-and low-risk groups were also analyzed. The GSE20685 dataset was used to validate the prognostic model.

### Analysis of immune cell abundance

To evaluate the fractions of 22 infiltrated immune cells in the high- and low-risk breast cancer groups, a deconvolution algorithm CIBERSORT using support vector regression was used based on gene expression profiles. We use the corresponding R package “CIBERSORT” to assess the immune cell abundance (27).

### TIMER database analysis

Tumor immune estimation resource (TIMER) database (https://cistrome.shinyapps.io/timer/), a comprehensive resource for systematic analysis of immune infiltrates across multiple cancer types, was applied to estimate tumor immune infiltration by B cells, CD4+T-cells, CD8+T-cells, dendritic cells, macrophages, and neutrophils immune infiltration data. Differences in the degree of infiltration between five gene copy number types (deep deletion, arm-level deletion, arm-level gain, diploid/normal, and high amplification) were assessed by TIMER database.

### Cell culture

MCF-7, MDA-MB-231, MCF-10A and T-47D cell lines were obtained from the Chinese Academy of Medical Sciences. MCF-7, MDA-MB-231, MCF-10A and T-47D cell lines were cultured with Dulbecco’s modified Eagle medium (DMEM) or Roswell Park Memorial Institute (RPMI) 1640 medium, were supplemented with 10% fetal bovine serum (FBS), 100 units/mL penicillin, and 100 mg/mL streptomycin (Thermo Fisher Scientific, Inc., Waltham, MA, USA). The cells were cultured in a humidified incubator equilibrated with 5% CO_2_ at 37°C.

### Real-time quantitative polymerase chain reaction analysis

To quantify the mRNA of target genes by RT-qPCR, total RNA was extracted from MCF-10A, T-47D, MCF-7, and MDA-MB-231 cells using TRIzol reagent. Reverse transcription was performed using the RevertAid First Strand cDNA Synthesis Kit (Roche, Basel, Switzerland) according to the manufacturer’s instructions. β-Actin was used as the internal reference. The relative expression of target genes was calculated using the 2^−ΔΔCt^ method.

### Clinical tissue samples

The paired breast cancer tissues and adjacent normal tissues were collected from six patients diagnosed with breast cancer at the Cancer Hospital Chinese Academy of Medical Sciences. Samples were collected and frozen in liquid nitrogen immediately after surgical and stored at −80°C. All the clinical samples were approved by the Ethics Committee of Cancer Hospital Chinese Academy of Medical Sciences, and informed consent was obtained from all patients. The clinical characteristics of the patients are shown in **Supplementary Table 2**.

### 5-ethynyl-2’-deoxyuridine incorporation assay

MDA-MB-231 cells with depletion of indicated genes and control cells were seeded into 6-well plates at a density of 0.8 × 10^5^ cells/ml to adhere overnight. After that, DNA proliferation was detected using an EdU assay kit according to the manufacturer’s instructions (RiboBio, Guangzhou, China).

### Cell invasion assay

Transwell chamber filters (BD Bioscience, San Jose, CA, USA) were coated with Matrigel diluted (1:10) in serum-free medium. MDA-MB-231 cells transfected with indicated siRNAs and control cells were seeded into the upper chamber of the transwell chambers, and the chambers were transferred into 24-well plates containing 500 μl culture medium per well. After 20 h of incubation, cells in the upper chamber were fixed with 4% formaldehyde, washed with PBS, and stained with crystal violet for half an hour. Images of invasive cells were captured using a light microscope.

### Western blot

Total proteins were separated by 10% SDS-PAGE gels. The proteins were transferred onto the PVDF membrane, then blocked with 5% non-fat milk. After that, the PVDF membrane was cropped according to the molecular weight of target proteins, followed by immunoblotting with the indicated antibodies: anti-Fibronectin, anti-Vimentin and anti-β-actin (Sigma Aldrich), anti-E-cadherin, anti-α-Catenin, anti-γ-Catenin and anti-N-cadherin (BD Bioscience). The β-actin content was analyzed as the loading control. Then the membranes were washed with PBST buffer for 3 times (5min/time), followed by incubation with secondary antibodies. After washing, the membranes were placed on an X-ray radiographic cassette, developed with ECL Chemiluminescent Western blot reagents and finally blotted onto X-ray films.

### Statistical analysis

All statistical analyses and corresponding visualization were performed using the R Studio software 3.6.3 (RStudio, Boston, MA, USA) and SPSS Statistics software (SPSS, Inc., Chicago, IL, USA). Statistical data were analyzed by Student’s t-test. All experimental data were analyzed and visualized with R Studio or GraphPad Prism 8 (GraphPad Software, Inc, San Diego, CA, USA). Kaplan-Meier curve analyses were performed using the “survminer” R package (https://cran.r-project.org/web/packages/survminer/index.html). For all statistical tests, two-tailed *P* < 0.05 denoted statistical significance, which is indicated by * *P* < 0.05, ** *P* < 0.01, *** *P* < 0.001, or **** *P* < 0.0001.

## Results

### TMB calculation and correlation with clinical parameters

**Figure 2:**
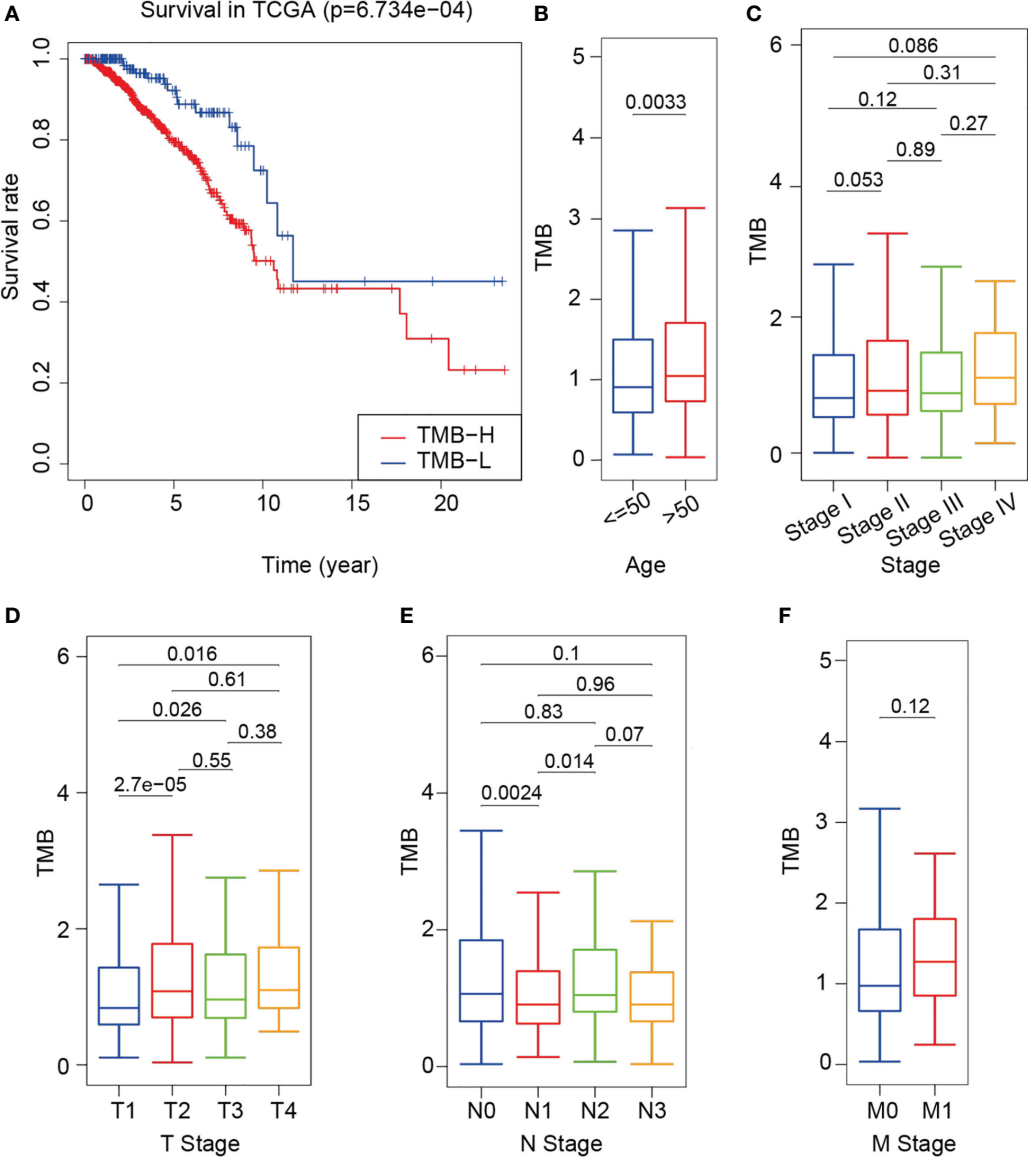
Difference in survival between the high- and low-TMB groups and correlation between TMB and clinical indexes. **(A)** Survival curve of training cohort based on TMB. **(B–F)** Correlational analyses between TMB and patient age, clinical stage, T stage, N stage and M stage. Student’s t-test.

Breast cancer genomic and transcriptomic data were obtained from a total of 960 tumor samples. The TMB value of each sample was calculated to evaluate the correlation between TMB and the clinical parameters. The survival rate of the high-TMB group was significantly lower than that of the low-TMB group (*P* = 6.734e-04) (**Figure 2A**). As shown in **Figures 2B, C**, TMB was significantly higher in older patients than in younger ones (*P* = 0.0033); however, TMB was not significantly different in terms of each clinical stage between older and younger patients (*P* > 0.05). TMB was significantly different between the T1 and T2 groups, the T3 and T4 groups (*P* < 0.05) (**Figure 2D**), the N0 and N1 groups, and the N1 and N2 groups (*P* < 0.05) (**Figure 2E**). However, TMB was not significantly different between the M0 and M1 groups (*P* = 0.12) (**Figure 2F**).

### DDR gene set and mutational landscape

**Figure 3:**
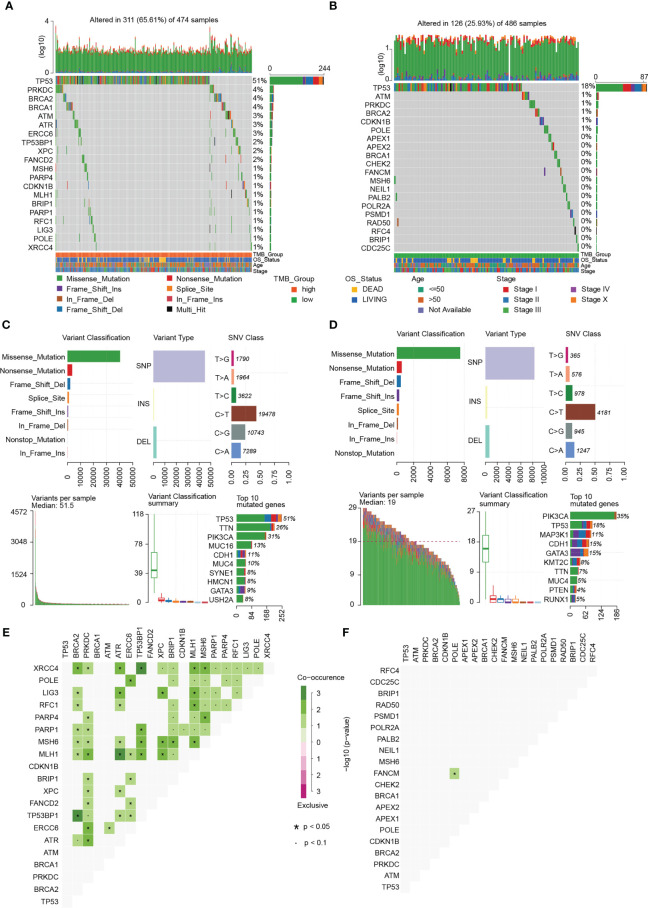
Mutational landscape of breast cancer. **(A)** Landscape of gene mutations of each high-TMB breast cancer sample in a waterfall plot. **(B)** Landscape of gene mutations of each low-TMB breast cancer sample in a waterfall plot. **(C)** Summary of statistical calculations for the frequency of mutation types in the high-TMB group. Missense mutation, single nucleotide polymorphism (SNP), and C > T mutation are the most frequent. Top 10 mutant genes in the high TMB group include *TP53*, *TTN*, *PIK3CA*, *MUC16*, *CDH1*, *MUC4*, *SYNE1*, *HMCN1*, *GATA3*, and *USH2A*. **(D)** Summary of statistical calculations for the frequency of mutation types in the low-TMB group. Missense mutation, SNP, and C > T mutation are the most frequent. Top 10 mutant genes in the low-TMB group include *PIK3CA*, *TP53*, *MAP3K1*, *CDH1*, *GATA3*, *KMT2C*, *TTN*, *MUC4*, *PTEN*, and *RUNX1*. **(E)** Co-occurrence and exclusive analyses in the high-TMB group. **(F)** Co-occurrence and exclusive analyses in the low-TMB group.

DDR gene sets were sorted out from 10 DDR-related signaling pathways, and a total of 193 DDR genes were obtained (21). The list of genes is shown in **Supplementary Table 3**. The landscape map of the DDR gene mutations in the high- and low-TMB group was visualized using the “maftools” R package (**Figure 3**). The top five high-frequency mutant genes in the high-TMB group were *TP53* (51%), *PRKDC* (4%), *BRCA2* (4%), *BRCA1* (4%), and *ATM* (3%). The top five high-frequency mutant genes in the low-TMB group were *TP53* (18%), *ATM* (1%), *PRKDC* (1%), *BRCA2* (1%), and *CDKN1B* (1%). It is important to note that 51% of the samples in the high-TMB group had a *TP53* mutant, while only 18% of the samples in the low-TMB group had a *TP53* mutant (**Figures 3A, B**). Overall, the high-TMB group had a higher frequency of gene mutations than the low-TMB group. In the high- and low-TMB groups, the median number of mutations was 51.5% and 19%, respectively. In addition, 90% of the mutations were point mutations (**Figures 3C, E**). Through the co-occurrence and exclusive analyses of these mutant genes, a total of 72 mutant gene pairs were obtained in the high-TMB group, and only one significant mutant gene pair was obtained in the low-TMB group (**Figures 3D, F**).

### Screening of differentially expressed DDR genes between the high- and low-TMB groups

**Figure 4:**
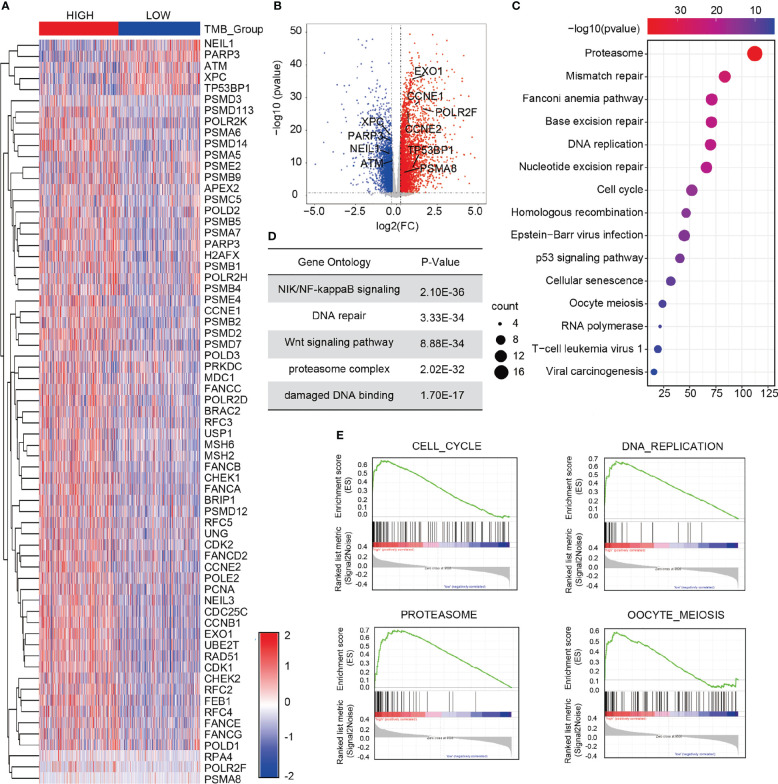
Screening of differentially expressed DDR genes of in the two TMB groups. **(A)** Heat map of 67 differentially expressed DDR genes in the high- and low-TMB groups. **(B)** Volcano plot of 6,424 differentially expressed genes in the high- and low-TMB groups. **(C)** KEGG analysis of the differential genes in breast cancer. **(D)** GO enrichment analysis of the differences in gene expression. **(E)** GSEA in the high- and low-TMB groups. Top four pathways enriched in the high-TMB group are cell cycle, DNA replication, proteasome, and oocyte meiosis.

A total of 6,424 differentially expressed genes was obtained between the high- and low-TMB groups. Compared with the low-TMB group, there were 2,686 genes up-regulated while 3,738 genes down-regulated in the high-TMB group, as shown in the volcano plot (**Figure 4A**). Among the 6,424 differentially expressed genes, there were 67 DDR genes. A series of oncogenes, including *EXO1*, *CCNE1*, *CCNE2*, *POLR2F*, *TP53BP1*, and *PSMA8*, was shown to be activated or inactivated (**Figure 4B**). Information of the 67 differentially expressed genes is listed in **Supplementary Table 4**. To further elucidate the molecular function of the differentially expressed genes in breast cancer, we performed Kyoto Encyclopedia of Genes and Genomes (KEGG) analysis and Gene Ontology (GO) enrichment analysis. KEGG analysis revealed that the differentially expressed DDR genes were mainly enriched in terms of the proteasome, mismatch repair, and homologous recombination (**Figure 4C**). The GO enrichment results revealed that the top five enriched signaling pathways of the 67 differentially expressed genes were the Nuclear factor kappa B (NF-κB) inducing kinase (NIK)/NF-κB signaling pathway, DNA repair pathway, Wnt signaling pathway, proteasome complex pathway, and damaged DNA binding pathway (**Figure 4D**). Furthermore, Gene Set Enrichment Analysis (GSEA) revealed that in the high-TMB group, the differentially expressed genes were mainly enriched in terms of the cell cycle, DNA replication, proteasome, and oocyte meiosis (**Figure 4E**).

### Screening of prognostic factors by univariate Cox Proportional hazard regression analysis

**Figure 5:**
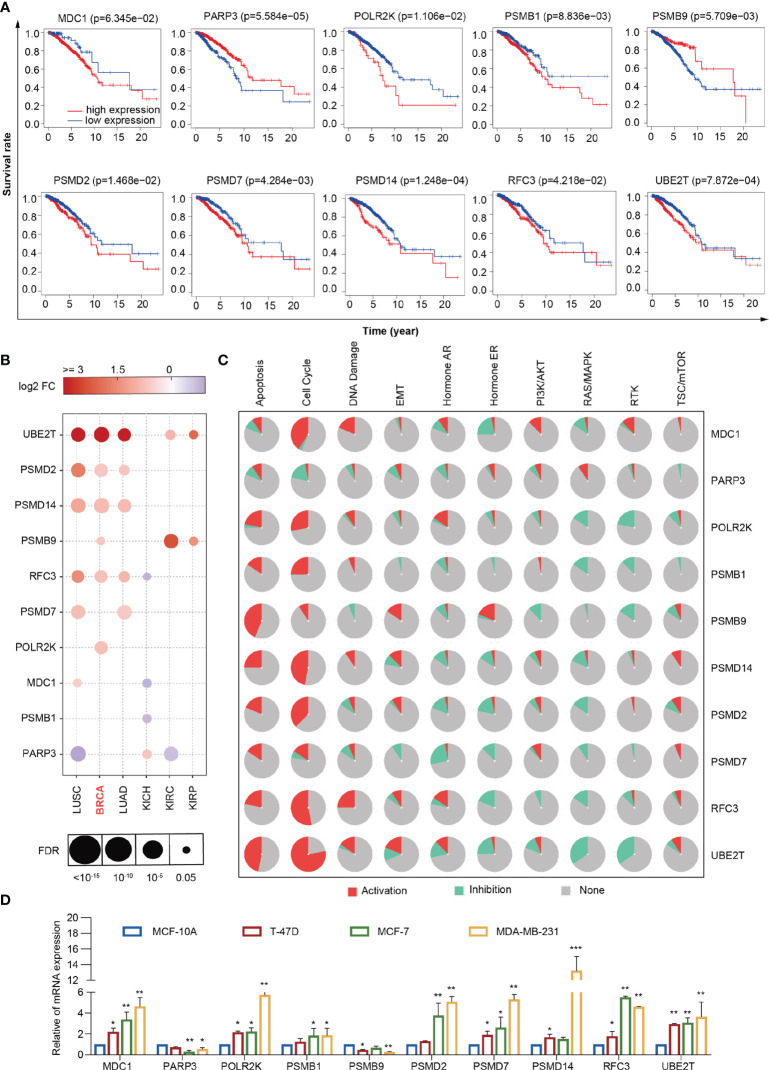
Comprehensive analysis of 10 DDR genes. **(A)** Kaplan–Meier analysis of *MDC1*, *PARP3*, *POLR2K*, *PSMB1*, *PSMB9*, *PSMD2*, *PSMD7*, *PSMD14*, *RFC3* and *UBE2T*. **(B)** Expression of *MDC1*, *PARP3*, *POLR2K*, *PSMB1*, *PSMB9*, *PSMD2*, *PSMD7*, *PSMD14*, *RFC3* and *UBE2T* in lung squamous cell carcinoma, breast cancer, lung adenocarcinoma, kidney chromophobe, kidney renal clear cell carcinoma, and kidney renal papillary carcinoma from the GSCALite database. **(C)** Different pathways correlated with *MDC1*, *PARP3*, *POLR2K*, *PSMB1*, *PSMB9*, *PSMD2*, *PSMD7*, *PSMD14*, *RFC3* and *UBE2T* from the GSCALite database. **(D)** RT-qPCR analyses of *MDC1*, *PARP3*, *POLR2K*, *PSMB1*, *PSMB9*, *PSMD2*, *PSMD7*, *PSMD14*, *RFC3* and *UBE2T* expressions in MCF-10A, T-47D, MCF-7, and MDA-MB-231 cells. Bars represent the mean ± SD of triplicate cell cultures (*P < 0.05, **P < 0.01, ***P < 0.001). Student’s t-test.

To screen the genes related to prognosis, we performed univariate Cox Proportional hazard regression analysis using the 67 differentially expressed DDR genes. Ten prognosis-related genes were selected: *MDC1*, *PARP3*, *POLR2K*, *PSMB1*, *PSMB9*, *PSMD2*, *PSMD7*, *PSMD14*, *RFC3*, and *UBE2T*. Information of these 10 genes were listed in **Supplementary Table 5**. We divided the 960 breast cancer samples into high- and low-expression groups according to the median expression of the prognosis-related genes in the samples. We also performed Kaplan-Meier survival analysis using the 10 DDR genes in the high- and low-expression groups. The survival rates of the two groups were analyzed using the log-rank test. There was a significant difference in terms of the overall survival rate between the high- and low-expression groups. The high expression of *PARP3* and the low expression of *POLR2K*, *PSMB1*, *PSMD2*, and *PSMD14* significantly prolonged the survival time of patients, improving outcomes and reducing recurrence rates (*P* < 0.05) (**Figure 5A**). In addition, we explored the expression and related pathways of the 10 genes using the Gene Set Cancer Analysis (GSCALite) database. As shown in **Figure 5B**, the expression levels of the genes were partially enhanced in breast cancer and lung adenocarcinoma. *MDC1*, *POLR2K*, *PSMB1*, *PSMB9*, *PSMD2*, *PSMD7*, *PSMD14*, *RFC3*, and *UBE2T* were highly activated in the apoptotic and cell cycle pathways and reserved in the Ras/mitogen-activated protein kinase (MAPK) pathway (**Figure 5C**). To validate the expression of *MDC1*, *PARP3*, *POLR2K*, *PSMB1*, *PSMB9*, *PSMD2*, *PSMD7*, *PSMD14*, *RFC3*, and *UBE2T* in breast cancer cells, we achieved real-time quantitative polymerase chain reaction (RT-qPCR) to identify the mRNA levels of these 10 genes in T-47D, MCF-7, and MDA-MB-231 human breast cancer cells and MCF-10A mammary epithelial cells (normal cells) (**Figure 5D**). The expression levels of *MDC1*, *POLR2K*, *PSMB1*, *PSMD2*, *PSMD7*, *PSMD14*, *RFC3*, and *UBE2T* were higher in breast cancer cells than in normal cells. In contrast, the expression levels of *PARP3* and *PSMB9* were lower in breast cancer cells than in normal cells. These results are consistent with those of the survival analysis, indicating the suitability of the prognostic model based on the prognosis-related genes.

### Analysis and evaluation of the prognostic model for breast cancer

**Figure 6:**
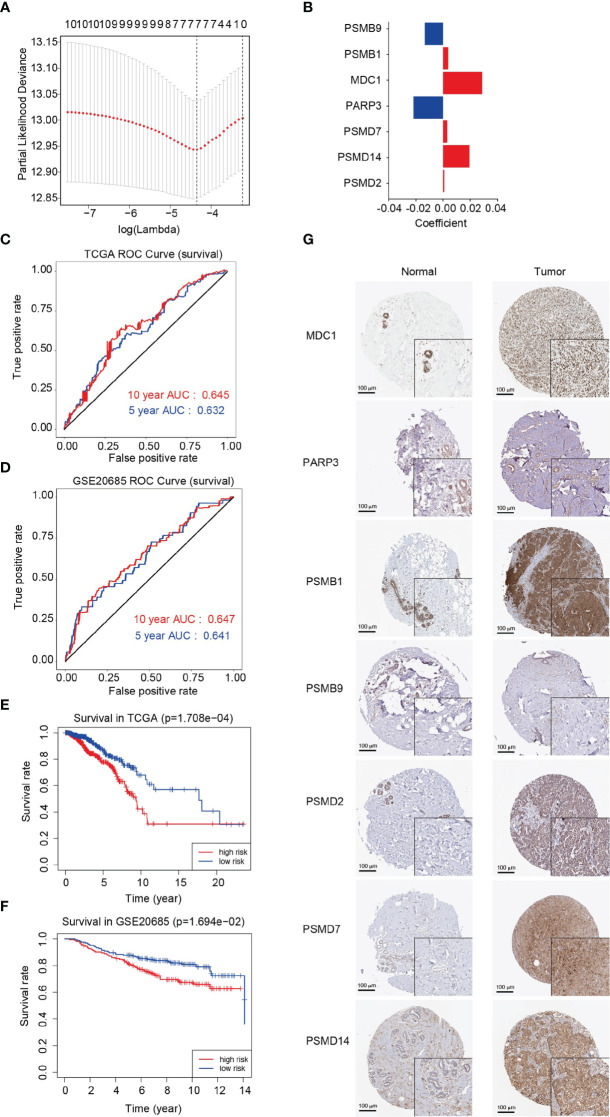
Analysis and evaluation of the prognostic model for breast cancer. **(A)** Determination of the Lambda coefficient of LASSO-Cox regression analysis. **(B)** The regression coefficients (coincident values) of the seven genes included in the model. **(C, D)** Area under the curve (AUC) of ROC curves displaying the predictive accuracy of the risk scores in the training and validation cohorts. **(E, F)** Kaplan-Meier analysis of the prognostic model in the training and validation cohorts. **(G)** Immunohistochemistry of the MDC1, PARP3, PSMB1, PSMB9, PSMD2, PSMD7 and PSMD14 proteins in breast cancer and normal breast tissues from the Human Protein Atlas (HPA).

The least absolute shrinkage and selection operator (LASSO)-Cox regression model was used to select the least redundant and most informative panel of genes to predict the prognosis of breast cancer. The parameter lambda.min was selected as the critical point for the linear risk assessment model composed of seven genes (*MDC1*, *PARP3*, *PSMB1*, *PSMB9*, *PSMD2*, *PSMD7*, and *PSMD14*) (**Figures 6A, B**). The gene descriptions and biological processes are shown in **Supplementary Table 6**. The median risk score of all patients was used as the cutoff value. The patients were divided into the high-risk (n = 480) and low-risk (n = 480) groups using the risk prognostic model to calculate the risk score of each patient in the training cohort. By analyzing the time-dependent receiver operating characteristic (ROC) curves, we discovered that the model exhibited a clinical significance in terms of the 5-year and 10-year survival rates in patients with breast cancer (AUC = 0.632 and 0.645, respectively), indicating the good prognostic ability of the model in breast cancer (**Figure 6C**). Additionally, Kaplan-Meier analysis displayed that the overall survival rate of patients in the high-risk group was significantly lower than that of patients in the low-risk group (*P* = 1.708e-04), indicating the suitability of the prognostic model for predicting the prognosis of patients with breast cancer (**Figure 6E**). After successfully establishing the prognostic model, we used the GSE26085 dataset as the validation cohort to analyze the overall survival rates and ROC curves. We found that the overall survival rate of patients in the high-risk group was significantly lower than that of patients in the low-risk group (*P* = 1.694e-02). We also found that the model exhibited a clinical significance in terms of the 5-year and 10-year survival rates (AUC = 0.641 and 0.647, respectively) in the validation cohort (**Figures 6D, F**). Subsequently, we explored the protein levels of MDC1, PARP3, PSMB1, PSMB9, PSMD2, PSMD7, and PSMD14 in normal breast tissues and breast cancer tissues. As shown in **Figure 6G**, the protein levels of MDC1, PSMB1, PSMB9, PSMD2, PSMD7, and PSMD14 were increased, while that of PARP3 was decreased in breast cancer tissues compared with normal tissues.

### Tumor-infiltrating immune cells in the prognostic model

**Figure 7:**
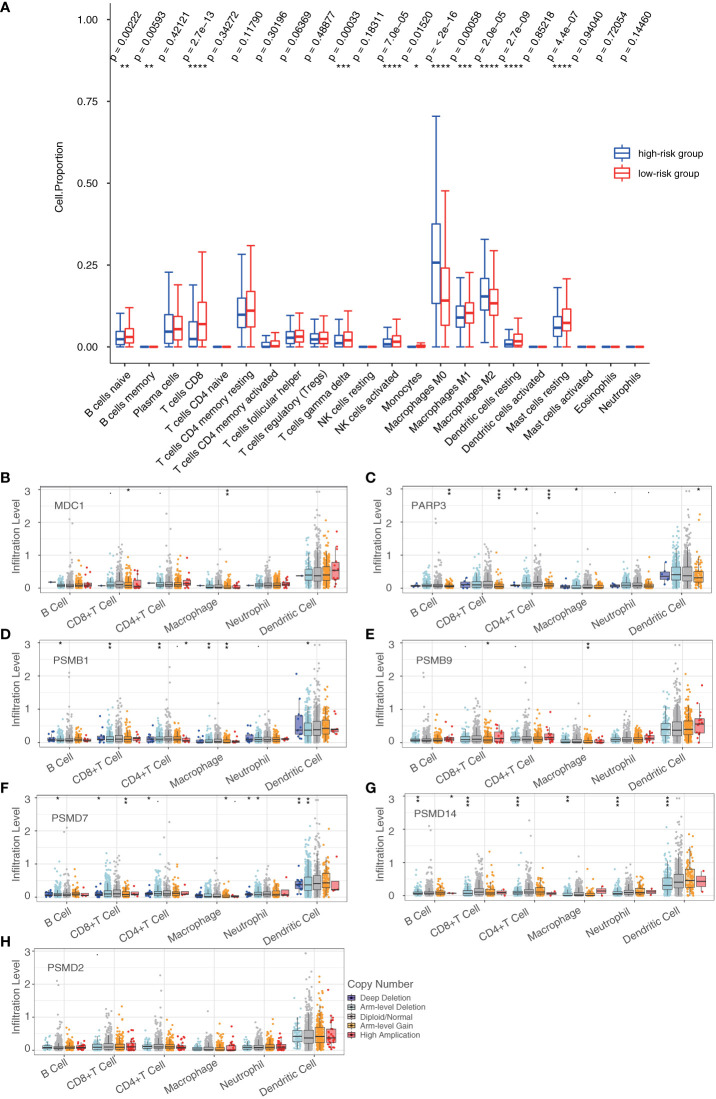
Tumor-infiltrating immune cells in the prognostic model. **(A)** Analysis of tumor-infiltrating immune cells in the high- and low-risk groups. **(B–H)** Analysis of the differences in immune infiltration and SCNAs among seven DDR genes (*P < 0.05, **P < 0.01, ***P < 0.001, ****P < 0.0001). Student’s t-test.

Then, we investigated 22 tumor-infiltrating immune cell subtypes in the high- and low-risk groups in the breast cancer training cohort. Of the subtypes, 11 varied significantly between the high- and low-risk groups. Furthermore, the CD8+ T cells, activated Natural Killer (NK) cells, M0 macrophages, M2 macrophages, resting dendritic cells, and resting mast cells showed significant differences in terms of expression between the high- and low-risk groups (*P* < 0.0001) (**Figure 7A**). Furthermore, we investigated the effects of the seven DDR genes on immune cell infiltration in the training cohort using the TIMER database. Different types of somatic copy number alterations, including those with deep deletion, arm-level deletion, arm-level gain, diploid/normal, and high amplification, in the seven genes were shown to significantly regulate immune cell infiltration in the breast cancer microenvironment (**Figures 7B–H**). Only the abundance of CD8+ T cells showed significant differences among all seven genes, indicating that CD8+ T cells may be a potential biomarker for distinguishing patients with favorable responses to immunotherapy based on our prognostic model.

### Functional identification of prognostic model in breast cancer

**Figure 8:**
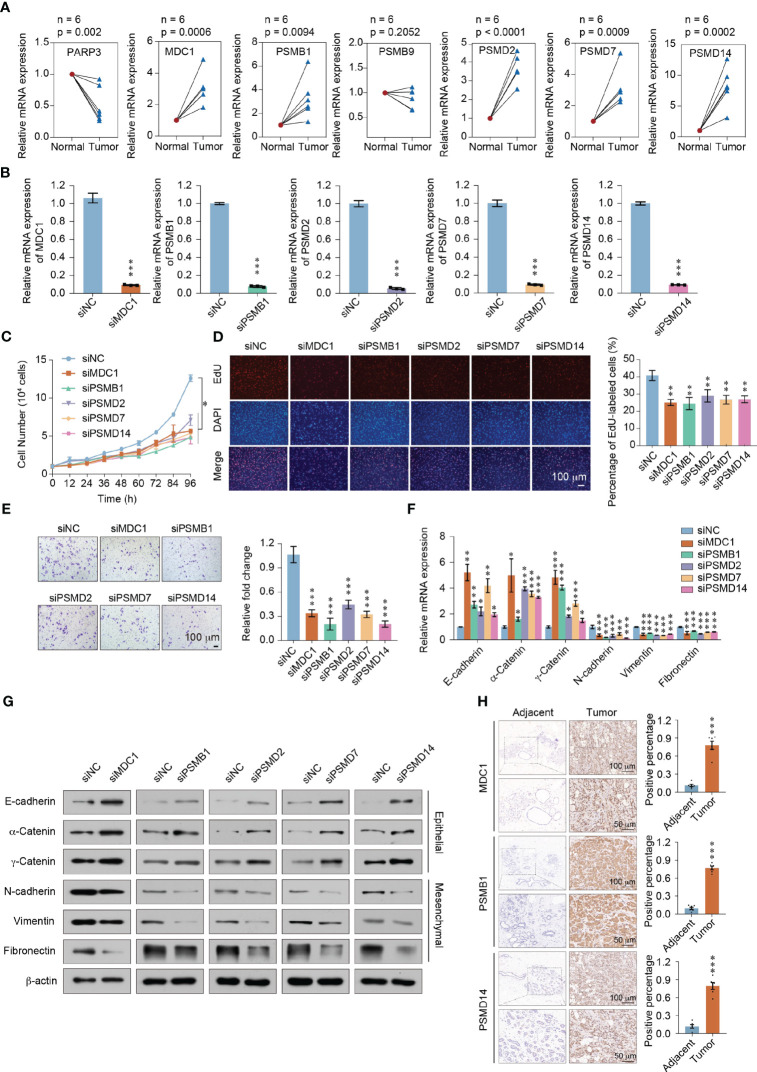
Functional identification of the prognostic model in breast cancer. **(A)** RT-qPCR analyses of *MDC1*, *PARP3*, *PSMB1*, *PSMB9*, *PSMD2*, *PSMD7*, and *PSMD14* expressions in 6 paired breast cancer tissues and adjacent normal tissues. **(B)** The knockdown efficiency was measured by qRT-PCR in MDA-MB-231 cells. **(C)** Growth curve assays were performed in MDA-MB-231 cells transfected with siRNAs against *MDC1*, *PSMB1*, *PSMD2*, *PSMD7*, or *PSMD14*. **(D)** EdU incorporation assays were performed in *MDC1*, *PSMB1*, *PSMD2*, *PSMD7*, or *PSMD14* depleted MDA-MB-231cells. Representative images are shown on the left, and statistical analysis is shown on the right. **(E)** Cell invasion assays were performed using the matrigel transwell filters in *MDC1*, *PSMB1*, *PSMD2*, *PSMD7*, or *PSMD14* depleted MDA-MB-231 cells. Invading cells were stained and counted. Representative images are shown on the left, and statistical analysis is shown on the right. **(F)** RT-qPCR analyses of EMT markers expressions in *MDC1*, *PSMB1*, *PSMD2*, *PSMD7*, or *PSMD14* depleted MDA-MB-231 cells. **(G)** Western blot using the indicated antibodies were performed on total protein extracted from *MDC1*, *PSMB1*, *PSMD2*, *PSMD7*, or *PSMD14* depleted MDA-MB-231 cells. **(H)** Immunohistochemical staining of MDC1, PSMB1, and PSMD14 in breast cancer and adjacent tissues (n = 6). Results were presented as mean ± SEM, two-tailed unpaired t test, ***P < 0.001. **(A–F)** Error bars represent means ± SD for three independent experiments (*P < 0.05, **P < 0.01, ***P < 0.001). Student’s t-test.

We first collected 6 paired breast cancer tissues and adjacent normal tissues to verify the mRNA expression of these 7 genes *in vivo*. Consistent with the Immunohistochemistry (IHC) results (**Figure 6G**), the expression of MDC1, PSMB1, PSMD2, PSMD7 and PSMD14 were significant augmented in breast cancer patients (*P* < 0.05). In contrast, the expression of PARP3 were significant declined in breast cancer patients (*P* < 0.05) (**Figure 8A**). Furthermore, we investigated the function of the five highly expressed genes (*MDC1*, *PSMB1*, *PSMD2*, *PSMD7*, and *PSMD14*) in breast cancer MDA-MB-231 cells to test the prognostic model. To this end, loss of function of the MDC1, PSMB1, PSMD2, PSMD7, and PSMD14 were first studied using growth curve assays. Small interfering RNA (siRNAs) targeting indicated genes were transfected into MDA-MB-231 cells (**Figure 8B**). As shown in **Figure 8C**, compared to the control, the cell proliferation was significantly inhibited when knockdown of each of the five genes (*P* < 0.05). And the EdU assays further confirmed the results of the growth curve assays (**Figure 8D**). In addition, we investigated the roles of MDC1, PSMB1, PSMD2, PSMD7, and PSMD14 in the invasion and metastasis of breast cancer. The cell invasion assays performed in MDA-MB-231 cells showed that knockdown each of the five genes decreased in the invasive potential of breast cancer cells (**Figure 8E**). The expression changes of Epithelial-Mesenchymal transition (EMT) markers in MDA-MB-231 cells harboring knockdown of *MDC1*, *PSMB1*, *PSMD2*, *PSMD7*, or *PSMD14*. Consistently, the results showed that depletion each of the five genes led to increased expression of epithelial markers including E-cadherin, α-Catenin and γ-Catenin at both mRNA and protein level, whereas expression of mesenchymal markers including N-cadherin, vimentin and fibronectin were downregulated (**Figures 8F, G**). These results suggested that in breast cancer patients, the highly expressed genes, including *MDC1*, *PSMB1*, *PSMD2*, *PSMD7*, and *PSMD14* are necessary to promote the proliferative state, invasion potential, and EMT of breast cancer cells. To further confirm the role of the prognostic genes, we focused on MDC1, PSMB1 and PSMD14, as they showed a significance effect in the previous set of experiments. Six paired breast cancer tissues and adjacent normal tissues to verify the protein expression level of MDC1, PSMB1 and PSMD14 via immunohistochemical staining. We found that the expression of MDC1, PSMB1 and PSMD14 were concurrently upregulated in breast cancer samples, suggesting the potential of the prognostic model (**Figure 8H**).

## Discussion

TMB can be used to forecast the efficacy of the immune checkpoint blockade (ICB) therapy and has become a useful biomarker for recognizing patients with cancer who will benefit from immunotherapy, including breast cancer (28, 29). According to a gene expression-based study, higher TMB was associated with poorer survival outcomes in HER2+ breast cancer (30). Multiple types of tumors with high TMB received positive feedback with atezolizumab monotherapy, demonstrating activity for agents targeting PD-1 and PD-L1 in advanced TMB high solid tumors (31). Besides, TMB has already been approved as a companion diagnostic biomarker for pembrolizumab (32).

However, research on the use of DDR genes from tumors with a high TMB to construct a risk model for cancer treatment and prognosis is currently limited. In this study, we employed Cox regression analysis and LASSO-Cox regression analysis to construct a linear risk assessment model using differentially expressed DDR genes between the high- and low-TMB groups based on the genomic and transcriptomic data and clinical information of the verification cohort. With such stratification strategies, clinicians would be able to conveniently personalize medical treatment and health management for each patient with breast cancer.

Several studies have investigated the use of TMB in identifying patients who may respond to immunotherapy (33, 34). In recent years, such studies have mapped and characterized changes in TMB in pathological mechanisms. One study evaluated the distribution of TMB in 100,000 cancer cases and found that a group of patients exhibited a high TMB, which was associated with microsatellite instability. A group of somatic mutations in the *PMS2* gene promoter was also identified to 10% of skin cancers and was significantly correlated with an increase in TMB (35). Furthermore, another study reported that the tumor types with the highest percentage of mutations were thyroid cancer, breast cancer, and melanoma (36). ICB therapy produces a lasting anti-tumor effect in a variety of cancers, but not all patients respond to this therapy. The evaluation of more than 300 patient samples of 22 different cancer types in four major clinical trials showed that TMB and T cell inflammatory gene expression profiles play a joint predictive role in distinguishing responders and non-responders to PD-1 antibody therapy (37). Clinical studies that investigated TMB discovered that TMB was significantly associated with the wild-type epidermal growth factor receptor (*EGFR*) gene and a *TP53* mutation-positive status in 92 patients with lung cancer who endured surgery between 2013 and 2016 (38). Therefore, analyzing specific genetic changes, such as in *TP53*, may be a useful alternative in predicting TMB.

In this study, we mapped the landscape of DDR gene mutations in the high- and low-TMB groups and found that the top five high-frequency mutant genes in the high-TMB group were *TP53*, *PRKDC*, *BRCA2*, *BRCA1*, and *ATM*. Moreover, we found that the frequency of gene mutations in the high-TMB group was higher than that in the low-TMB group. *TP53* is known to have a major role in the regulation and repair of genomic damage. *TP53* gene mutations are one of the most common mutations in several cancers. There is considerable evidence proving that *TP53* affects TMB. Patients with *TP53* mutations represent a different molecular cohort that exhibits a poor prognosis. It was discovered that the expression of PD-L1 was enhanced in the hematopoietic stem cells of patients with *TP53* mutations, which were related to the upregulation of MYC and downregulation of the p53 transcriptional target miR-34a. It is worth noting that patients with TP53 mutations showed a significant decrease in the number of bone marrow infiltrating T cells, leading to a decrease in ICOS+ and 4-1BB + natural killer (NK) cells (39). Moreover, the microenvironment of *TP53*-mutant myelodysplastic syndromes (MDS) has been shown to possess immune-dominant and immune-evasive phenotypes, which may provide better therapeutic effects for patients with such *TP53* mutations. *BRCA1*/2 alterations are caused by somatic or germline mutations or homologous recombination (HR)-related defects caused by other factors (40). For instance, *BRCA1* promoter methylation or other potential mutations in DDR genes can lead to *BRCA1*/2 deficiency in patients with breast cancer (41). The combination therapy of cisplatin and PD-1 significantly enhanced the anti-tumor immunity of *BRCA1*-deficient mice, resulting in a strong systemic and intratumoral immune response (42). Furthermore, triple-negative breast cancer (TNBC) with *BRCA1* mutations treated with ICB therapy was reported to improved clinical outcomes. Ultimately, *TP53*, *PRKDC*, *BRCA2*, *BRCA1*, and *ATM* mutations may be potential biomarkers for predicting the clinical response of patients to immunotherapy to improve breast cancer survival.

We analyzed a prognostic model based on seven DDR genes, *PSMD2*, *PSMD7*, *PSMD14*, *PARP3*, *MDC1*, *PSMB1*, and *PSMB9*, reflecting an enhanced level of predicting the survival and prognosis of patients with breast cancer. According to our enrichment analysis, *PSMD2*, *PSMD7*, *PSMD14*, *PSMB1*, and *PSMB9* were involved in proteasome complex, *MDC1* and *PARP3* were involved in DNA repair signaling pathway. The 26S proteasome non-ATPase regulatory subunit (PSMD) 2 (PSMD2), PSMD7, and PSMD14 proteins participate in the ubiquitin-proteasome system, which plays a potential role in the proliferation and progression of tumor cells. In hepatocellular carcinoma cells, *PSMD2* knockout reduced the formation of lipid droplets and modulated the expression of genes associated with lipid synthesis through the p38-JNK and AKT signaling pathways (43). PSMD7 has similar functions in breast cancer. The level of PSMD7 was significantly elevated in breast cancer and was closely related to tumor subtype, tumor size, lymph node invasion, and tumor-node-metastasis (TNM) stage. PSMD14 participates in the regulation of cancer occurrence and progression through a variety of molecular mechanisms. In our study, the *PARP3*, *POLR2K*, *PSMB1*, and *PSMD2* genes were significantly associated with the overall survival of patients with breast cancer, and the high expression levels of *POLR2K*, *PSMB1*, and *PSMD2* were related to low survival rates. Proteasome β-subunit 1 (PSMB1), a member of the proteasome β-subunit family, was found to interact with inhibitor of IκB kinase ϵ (IKK-ϵ) and promote the degradation of IKK-ϵ. The binding of PSMB1 to the *BCL*-3 oncogene is necessary for proteasome degradation. As such, cells with a *PSMB1* deletion were found to exhibit defects in the polyubiquitin degradation of the BCL-3 protein (44). In addition, PSMB1 was shown to affect the response of follicular lymphoma to bortezomib and that the presence of the *PSMB1* rs12717 minor allele predicted the ineffectiveness of bortezomib in patients with myeloma (45). Based on our results, combined with the above evidence, our seven-gene prognostic model has the prospective ability to predict the survival and prognosis of patients with breast cancer. Moreover, three DDR genes, *POLR2K*, *PSMB1*, and *PSMD2*, which are closely linked to tumorigenesis, may be used as potential biomarkers for predicting the prognosis of patients with breast cancer.

During cancer treatment, an effective immune response is required to damage the function of tumor cells and ultimately destroy them (46). However, tumor cells have evolved a variety of mechanisms to escape immune surveillance, resulting in the inhibition of immune cell function and loss of the anti-tumor immune response (47, 48). Therefore, a new type of monoclonal antibody, ICIs, has become one of the most critical immunotherapeutic methods in cancer treatment (49, 50). Advances in glycomics have unveiled several cancer-specific glycosignatures, which provides a clinical-translational platform for glycomic studies towards precision medicine (51). Cancer vaccines developed from neoantigens are also a therapeutic anti-cancer immune responses. Novel strategies where tumourassociated carbohydrate antigens (TACAs) target glycan binding receptors (GBRs) on the surface of antigen presenting cells (APCs) can boosting immune responses (52). In immunotherapy research, analysis of immune cell infiltration in cancer is necessary. So, we discovered that there were significant differences in terms of immune cell abundance, particularly in terms of CD8+ T cells, activated NK cells, and M0, M1, and M2 macrophages, between the high- and low-TMB groups. CD8+ T cells can produce and express T cell receptors in the thymus. T cells induce immune responses in cancer, autoimmunity, and infection, and Th cells and CD8+ T cells play an essential role in tumor progression. In a study involving mice with TNBC, memory CD8+ T cells were found to be improved in the peripheral blood (53). In another study, the effect of *Plasmodium* infection on mouse breast cancer cells was determined to be linked to the initiation of an anti-tumor immune response regulated by CD8+ T cells (54). In addition, an analysis of clinical samples of metastatic melanoma revealed that the coexistence of CD20+ B cells and CD8+ T cells in tumors was related to the improvement of survival of patients with metastatic melanoma. Thus, CD8+ T cells likely play a key role in the immune microenvironment of melanoma, improving clinical outcomes, and may predict the prognosis of patients subjected to ICB therapy (55). NK cells, which also play a main role in immunity, are not only involved in immunoregulation and anti-tumor and anti-viral infection responses, but also participate in hypersensitivity and autoimmunity on certain occasions. In a TNBC xenotransplantation model, the distribution and aggregation patterns of NK cells in the tumor site were found to differ across the distinctive stages of tumor progression (56).

There are many events of gene copy number variation in the progression of breast cancer. In this study, we analyzed SCNAs and immune cell infiltration to assess the function of our prognostic model based on seven genes. The SCNAs of *PARP3*, *PSMB1*, *PSMD7*, and *PSMD1* showed significant differences in terms of immune infiltration, particularly in terms of B cell, CD8+ T cell, CD4+ T cell, macrophage, and dendritic cell infiltration. Poly (ADP-ribose) polymerase (PARP) 3 (PARP3) exhibits high homology with PARP1 and PARP2. PARP3 plays a role in DNA single- and double-strand break repair and humoral immunity. PARP3 was reported to be associated with the progression of gliomas and breast cancer. Moreover, the inhibition of PARP3 in lung cancer cells and osteosarcoma cells was found to increase telomerase activity, promote telomere maintenance, and lessen gene instability (57). Similarly, the absence of PARP3 was reported to enhance NADPH oxidase 4 (NOX4)-induced oxidative stress and reduce mechanistic target of rapamycin complex 2 (mTORC2) activation, resulting in an inefficient differentiation of neural stem cells or progenitor cells into astrocytes after birth (58). PARP3 was also found to interact with glycogen synthase kinase 3 beta (GSK3 β), a positive regulator of ubiquitin and rapamycin-insensitive companion of mammalian target of rapamycin (RICTOR) degradation, producing adenosine diphosphate. Knockout or inhibition of the *PARP3* gene aggravated centrosome amplification and genomic instability, reducing the proliferation, survival, and tumorigenicity of *BRCA1*-deficient TNBC cells (59). These results suggest that targeting the catalytic activity of PARP3 is a suitable approach for the treatment of *BRCA1*-related tumors through the RICTOR/mTORC2 signaling pathway. Currently, research on PARP3 and the tumor immune microenvironment is still limited. Our results suggest that focusing on PARP3, PSMB1, PSMD7, and PSMD14 and their roles in immunotherapy is a reasonable strategy for breast cancer treatment.

## Conclusions

In summary, we established and validated a seven-gene prognostic model based on TMB characteristics and DDR genes and showed that the model has potential applications in predicting the clinical benefits of ICB therapy and the prognosis of patients with breast cancer. This model can also be used to determine patients with breast cancer who would respond favorably to immunotherapy. The limitation of this study is that the molecular mechanisms of the DDR genes were not fully explored. Therefore, prospective studies are needed to verify our seven-gene prognostic model and to further elucidate the detailed molecular mechanisms of the seven DDR genes as clinical biomarkers.

## Data availability statement

Publicly available datasets were analyzed in this study. This data can be found here: The GSE20685 dataset from the GEO database (https://www.ncbi.nlm.nih.gov/geo/).

## Ethics statement

All the clinical samples used in this study were approved by the Ethics Committee of Cancer Hospital Chinese Academy of Medical Sciences, and informed consent was obtained from all patients. All the experiment protocol for involving human data was in accordance with the guidelines of national/international/institutional or Declaration of Helsinki in the manuscript.

## Author contributions

XT and WH contributed conception and designed the research study. BY, YY, JL, XW, XY, TM, and HY collected and organized the database. XT, TY, BY, and YY performed data analysis. TY, BY, YY, and SW performed the validation experiments. TY and WH wrote the manuscript. XT, BY, YY, JL, XW, and YW revised the manuscript. All authors read and finally approved the manuscript. All authors contributed to the article.
